# Correlation Assessment between Three-Dimensional Facial Soft Tissue Scan and Lateral Cephalometric Radiography in Orthodontic Diagnosis

**DOI:** 10.1155/2016/1473918

**Published:** 2016-05-29

**Authors:** Piero Antonio Zecca, Rosamaria Fastuca, Matteo Beretta, Alberto Caprioglio, Aldo Macchi

**Affiliations:** Department of Surgical and Morphological Sciences, University of Insubria, 21100 Varese, Italy

## Abstract

*Purpose*. The aim of the present prospective study was to investigate correlations between 3D facial soft tissue scan and lateral cephalometric radiography measurements.* Materials and Methods*. The study sample comprised 312 subjects of Caucasian ethnic origin. Exclusion criteria were all the craniofacial anomalies, noticeable asymmetries, and previous or current orthodontic treatment. A cephalometric analysis was developed employing 11 soft tissue landmarks and 14 sagittal and 14 vertical angular measurements corresponding to skeletal cephalometric variables. Cephalometric analyses on lateral cephalometric radiographies were performed for all subjects. The measurements were analysed in terms of their reliability and gender-age specific differences. Then, the soft tissue values were analysed for any correlations with lateral cephalometric radiography variables using Pearson correlation coefficient analysis.* Results*. Low, medium, and high correlations were found for sagittal and vertical measurements. Sagittal measurements seemed to be more reliable in providing a soft tissue diagnosis than vertical measurements.* Conclusions*. Sagittal parameters seemed to be more reliable in providing a soft tissue diagnosis similar to lateral cephalometric radiography. Vertical soft tissue measurements meanwhile showed a little less correlation with the corresponding cephalometric values perhaps due to the low reproducibility of cranial base and mandibular landmarks.

## 1. Introduction

Skeletal and dental components are of great importance in craniofacial diagnosis and orthodontic treatment planning [1]. Hard tissue is routinely evaluated by means of lateral cephalometric radiography collected by clinicians prior to orthodontic therapy. Besides skeletal evaluation, facial soft tissue analysis is assuming a relevant role in orthodontic diagnosis and treatment planning, since clinicians need to carefully assess the effects of dental and skeletal changes on the soft tissue profile when managing orthodontic treatment in order to estimate facial changes along with occlusal improvements [2]. Therefore, soft tissue analysis might represent an important source of treatment outcome evaluation and additional information for diagnosis.

Although cephalometric analysis of lateral radiography is spreading among orthodontists, its role in diagnosis and treatment planning is still debated [3]. Moreover, the fundamental* principles of justification*, optimization, and dose limitation should always be considered when radiographic examinations are performed at the beginning of the orthodontic treatment.

The growing interest in noninvasive diagnosis has allowed the development of new imaging tools which could enhance the role of soft tissue in diagnosis. Nevertheless, the difficulty of performing facial examinations reliably is probably responsible for the secondary role of soft tissue analysis in supporting diagnosis compared with skeletal analysis [4, 5].

Several analyses have been proposed for the evaluation of facial soft tissue. Most of them include photographic images in lateral position [6–9]. Some authors [4, 5] have also proposed soft tissue evaluation with frontal pictures and underlined the importance of reproducible head position during image acquisition.

Among the recently introduced noninvasive imaging techniques, stereophotogrammetry and laser scanning allow accurate acquisition of three-dimensional (3D) facial soft tissue with the possibility of locating landmarks and measuring angles, distances, surfaces, and volumes [10–13]. Even though normative values of 3D facial soft tissue are not available for the general population, some proposals have been made for specific sagittal and vertical measurements [14, 15] but further studies are needed to improve their reliability.

The difficulties related to the use of the appropriate equipment and software and the absence of reliable normative values for 3D facial soft tissue measurements might prevent their adoption by clinicians, who are still using lateral cephalometric radiography to perform their diagnosis.

The relationships between facial soft tissue and underlying hard tissue should be considered and investigated for any correspondences that might improve noninvasive orthodontic diagnosis and thus reduce patients' exposure to ionizing radiation.

The soft tissue profile may reflect the underlying skeletal and hard tissue, and it would be possible to estimate the skeletal configuration by visual inspection of the soft tissue profile alone, as suggested by previous studies [16]. Validation of the anatomy of facial soft tissue is fundamental for an objective analysis of craniofacial morphologies.

The aim of the present study was therefore to investigate correlations between facial soft tissue scans and lateral cephalometric radiography measurements.

## 2. Materials and Methods

### 2.1. Patient Selection

Signed informed consent to the release of diagnostic records for scientific purposes was obtained from patients prior to enrolment in the present prospective study. The protocol was reviewed and approved by the Ethical Committee (Approval number 6154) and procedures followed adhered to the Declaration of Helsinki. The final study sample comprised 312 subjects: 155 males (mean age of 24.3) and 157 females (mean age of 25.8). Inclusion criteria were Caucasian ethnic origin, age between 20 and 30 years to avoid errors arising from soft tissue laxity which might increase with age, and normal body mass index (BMI) [17]. Subjects were selected from those patients seeking orthodontic treatment for whom a diagnostic lateral cephalometric radiograph had been recorded within the previous six months. Exclusion criteria were craniofacial syndromes or anomalies, noticeable asymmetries, and previous or current orthodontic treatment that might affect the homogeneity of the sample.

**Figure 1:**
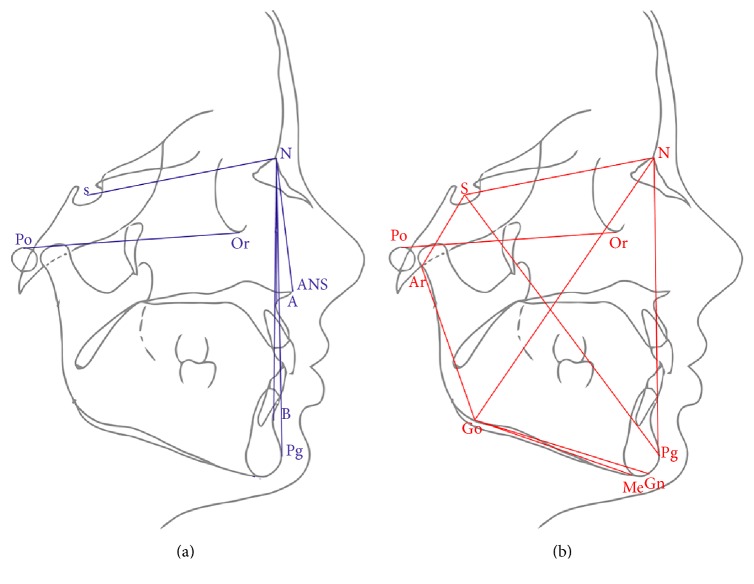
2D tracings and cephalometric analysis performed with lateral cephalometric radiography. (a) Sagittal measurements and (b) vertical measurements.

Lateral cephalometric radiographs were then collected for all subjects and cephalometric measurements were performed with Deltadent software (Outside Format, Milan, Italy) (Figure 1).

A facial scanner (Primesense Carmine 1.09, Subsidiary of Apple Inc., Israel, 2005) was employed for acquisition of the facial soft tissue of the subjects. The subject-to-scanner distance was set at 80 cm and scan time was 30 s on average. The scanner depth sensor data were 640 × 480 pixels. Data were recorded on a desktop workstation with a 2.6 GHz i7 Intel processor (Dell, Wicklow, Ireland).

Light conditions were set in order for reliable data capture. The subjects were seated with the lips relaxed and with the head in natural head position (NHP) (self-balance “mirror” position) as described by previous authors [18–20]. If a subject moved between scans, the procedure was repeated and the data of the first scan were eliminated from the study.

**Figure 2:**
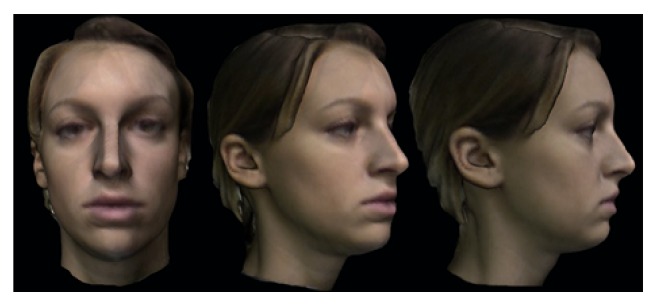
Facial soft tissue scan. Frontal, prospective, and right lateral 3D views of facial soft tissue scan of female patient.

The data were acquired by dedicated Skanect software (developed by the ManCTL Company, 2011, Madrid) (Figure 2). Mimics software (version 10.11, Materialise Medical Co., Leuven, Belgium) was used to import the surface model and to perform 3D cephalometric analysis.

All the lateral cephalometric radiographs underwent reposition of the head on the basis of the orientation of the soft tissue scan position by superimposition on the right lateral view of the 3D facial scan using Deltadent software.

**Figure 3:**
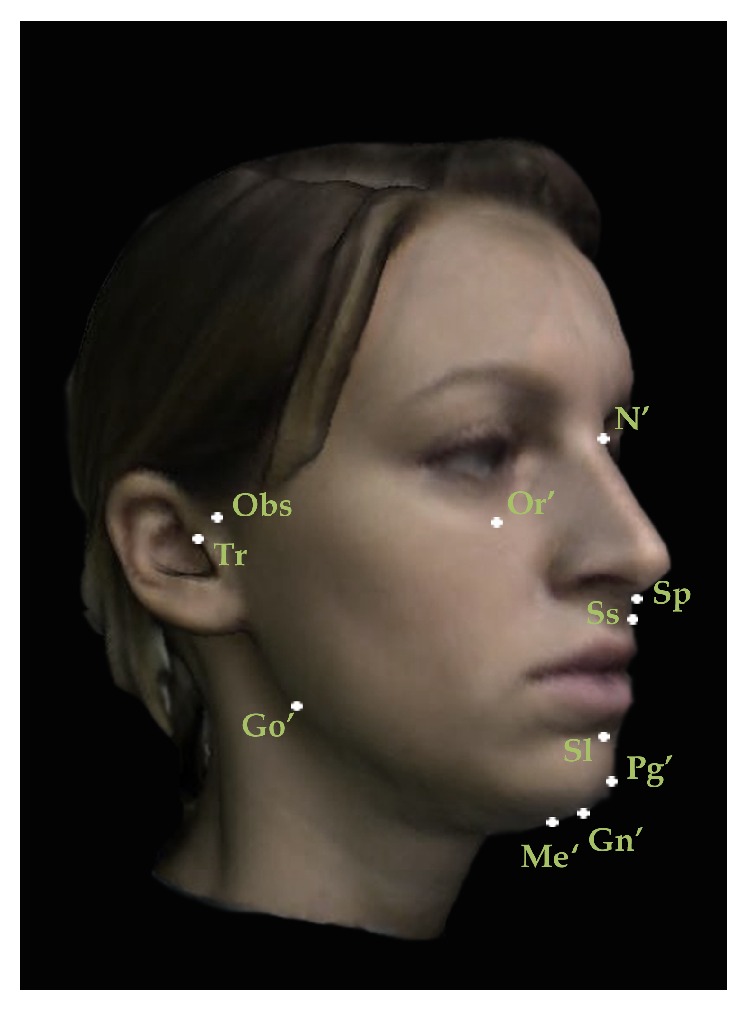
Set of reproducible landmarks employed to perform sagittal and vertical soft tissue 3D measurements: N′ Nasion; Obs Otobasion; Tr Tragus; Or′ Orbitale; Sp Spinal; Ss Subspinal; Go′ Gonion; Sl Sublabial; Pg′ Pogonion; Me′ Menton; Gn′ Gnation.

**Figure 4:**
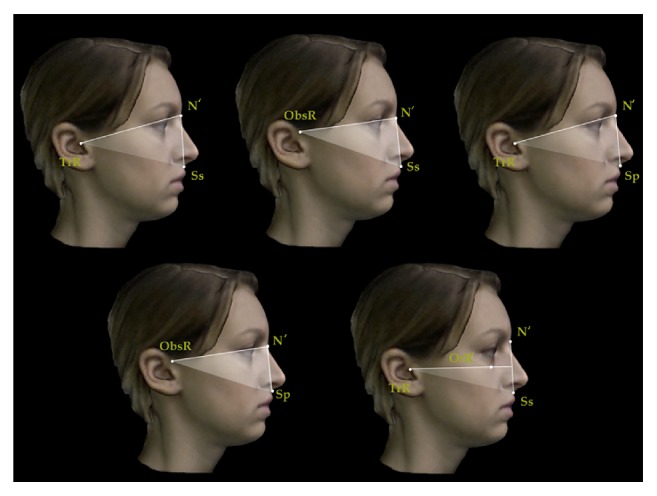
Sagittal angular measurements for 3D facial soft tissue. R: right. Maxillary sagittal measurements.

**Figure 5:**
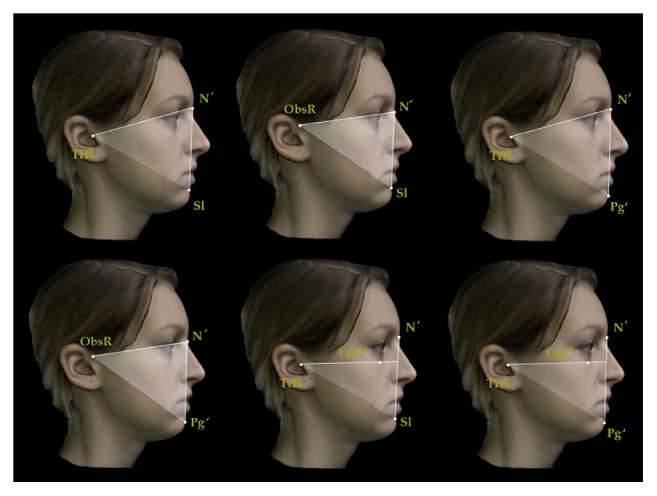
Sagittal angular measurements for 3D facial soft tissue. R: right. Mandibular sagittal measurements.

**Figure 6:**
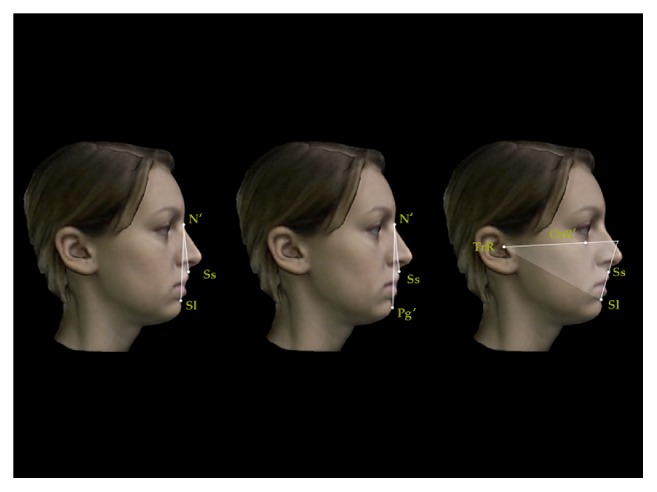
Sagittal angular measurements for 3D facial soft tissue. R: right. Maxillomandibular sagittal measurements.

**Figure 7:**
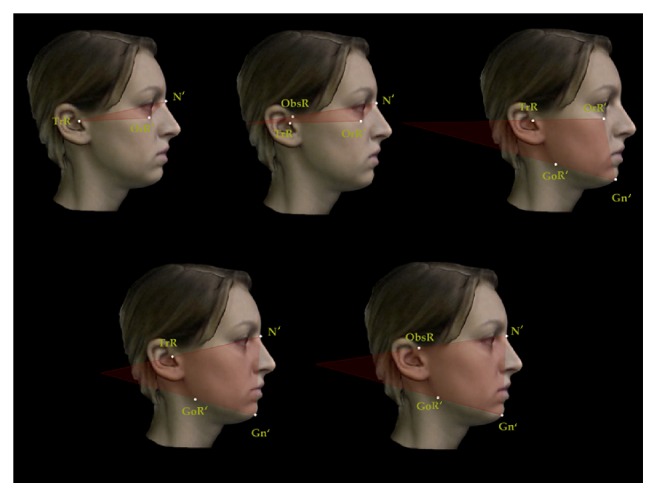
Vertical angular measurements for 3D facial soft tissue. R: right. Part 1.

**Figure 8:**
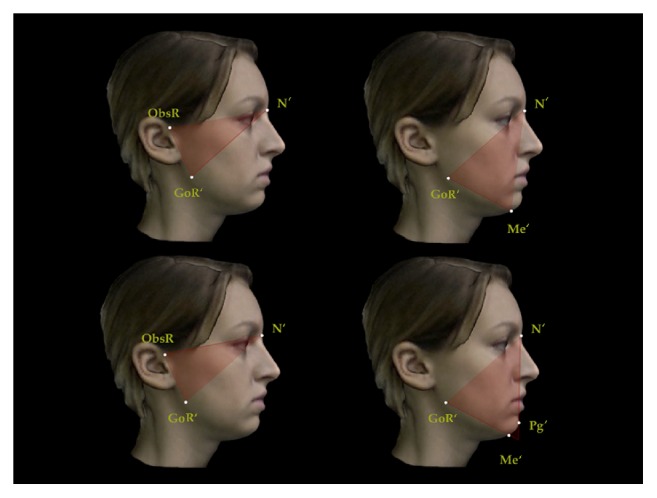
Vertical angular measurements for 3D facial soft tissue. R: right. Part 2.

**Figure 9:**
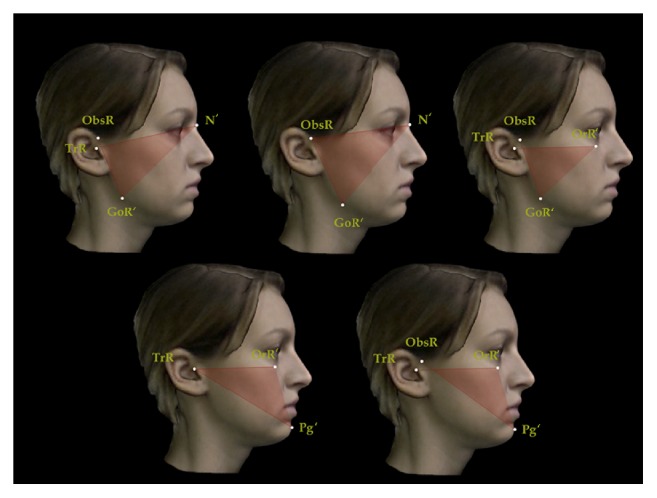
Vertical angular measurements for 3D facial soft tissue. R: right. Part 3.

A set of reproducible landmarks was developed to compute the soft tissue cephalometric analysis (Figure 3). Fourteen sagittal (Figures 4, 5, and 6) and 14 vertical (Figures 7, 8, and 9) angular measurements were selected and performed for good anatomical correspondence between hard tissue and soft tissue structures and reference landmarks. The average of the angles was computed for symmetric structures.

Then, every skeletal measurement was coupled and assigned to one or more soft tissue measurement (Table 1) for the correlation analysis.

### 2.2. Statistical Analysis

A pilot study was executed on 20 patients (12 males and 8 females) for the power analysis.

One sagittal and three vertical measurements were employed as main outcome for the power analysis as follows: SsN′Sl, TrOr^′∧^Go′Gn′, TrN^′∧^Go′Gn′, and ObsN^′∧^Go′Gn′. No differences in gender were included in the power analysis.

According to the power analysis, 300 subjects were required in order to obtain power of 0.80 for the present study.

SPSS software, version 22.0 (SPSS® Inc., Chicago, Illinois, USA), was used to run statistical analyses. The Shapiro-Wilk test revealed a normal distribution of tested variables. The mean and standard deviation (SD) of each of the variables were then calculated. Independent *t*-test was used to compare the mean differences between females and males and *P* < 0.05 was set as the level of significance. All the variables were then further analysed for any correlations with corresponding lateral cephalometric radiography measurements with the Pearson correlation coefficient (*r*) with the level of significance set at *P* < 0.05.

### 2.3. Method Error

All the measurements were performed by the same trained operator. Thirty of the 3D facial scans were repeated two weeks after the first recording and measurements were performed. The Dahlberg coefficient [21] was used to test the reproducibility of all the soft tissue landmarks employed. All the parameters displayed a method error < 1°, which is considered clinically irrelevant.

## 3. Results

Mean and standard deviations were calculated for each lateral cephalometric radiograph and soft tissue measurement.

The tested variables such as mean and SD did not show significant differences in terms of gender-specific differences (Table 2), and the following statistical analyses were performed for the total sample.

Medium, low, and high correlations were found for sagittal parameters and vertical parameters in assessment of correlation with the corresponding lateral cephalometric radiography measurements previously assigned (Tables 3 and 4).

ANB, ANPg, and FH^∧^AB were the only sagittal variables which showed high correlation coefficients compared with the respective soft tissue variables. Conversely, FH^∧^NA and SNans showed low correlation coefficients (Table 3).

No high correlations coefficients were found for the vertical parameters which showed medium correlation coefficients except for SN^∧^FH that exhibited low correlation compared with the respective soft tissue variables (Table 4).

## 4. Discussion

The purpose of this study was to compare facial soft tissue analysis, obtained from facial scans, with lateral cephalometric radiography, in order to highlight possible correspondences between hard tissue and soft tissue diagnoses. The growing role of noninvasive imaging tools could be of great importance in orthodontic diagnosis since 3D facial soft tissue might be employed as the first screening examination for guiding clinicians through skeletal diagnosis and performance of further radiological exams only when needed.

The tested infrared scanner showed good reliability and reproducibility in facial morphology acquisition. Moreover, the facial scans proved appropriate for landmark location and the method error for soft tissue cephalometric analysis was acceptable. The possibility of evaluating soft tissue components in 3D allowed us to relocate the head and did not present the limitation of bidimensional (2D) photographic pictures where head position errors can be of great importance in landmark identification.

The sample was first tested for any gender-specific differences. Kochel et al. [14] employed stereophotogrammetry for the evaluation of facial soft tissue focusing on sagittal measurements and found significant differences between males and females with the mean age (25.4 years) similarly to the sample in the present study. According to the present results, no significant differences in facial soft tissue sagittal and vertical dimensions were found between the genders, even though a tendency toward statistical significance (*P* < 0.08) was reported for TrN′Ss and ObsN′Ss measurements (Table 2). These measurements indicated maxillary protrusion and were reported to be smaller in females. Although similar samples were tested, the results of the present study were not in agreement with those of Kochel et al. [14]. Indeed, the methods of facial scanning and the soft tissue analysis employed should be considered as possible reasons for the different results in the variability of sagittal dimensions between genders. Moreover, in the present sample, gender differences seemed to have no influence on vertical dimensions in agreement with previous investigations [15].

Since no differences were assessed between genders, the sample was considered as a whole and Pearson correlation coefficients were computed for each variable in order to check for any correlations between sagittal and vertical soft tissue measurements and corresponding skeletal measurements in lateral cephalometric radiography. Little previous evidence has been presented for correspondences between soft tissue and skeletal measurements with 3D and 2D image acquisition tools [22, 23] but they showed high correlations between the tested variables. The present results were analysed for sagittal and vertical measurements, separately.

Most of the sagittal parameters showed medium correlation coefficients (between 0.31 and 0.67) (Table 3). ANB, ANPg, and FH^∧^AB showed high correlation coefficients (*r* > 0.7) when compared with the respective soft tissue variables. These angles are usually applied in the evaluation of sagittal relationships between maxilla and mandible and account for the diagnosis of skeletal malocclusion. According to the present results, the diagnosis performed on soft tissue seemed to be reliable in predicting skeletal cephalometric outcomes since the coefficients showed high values and reached the level of significance. Kochel et al. [14] evaluated sagittal soft tissue measurements and their correspondences with lateral cephalometric radiography, describing a set of variables defined on the basis of common skeletal cephalometric measurements. Their findings are in agreement with the present investigation. The selection of the corresponding landmarks between skeletal and soft tissue seemed very important in the outcomes of correlation coefficients. Previous studies [24] found no correlation between soft tissue measurements of facial profile and cephalometric ANB angle, employing the landmarks subnasal and skin pogonion as correspondent of skeletal landmarks A and B, respectively. The present investigation used the landmarks subspinal (Ss) and sublabial (Sl) and high correlation between soft tissue and ANB skeletal angle was found, showing that different outcomes might be owing to the selection of different landmarks.

The measurements of maxillary sagittal position (SNA, SNans, and FH^∧^NA) showed the lowest *r* values (ranging from 0.16 to 0.36) in relation to soft tissue corresponding measurements. On the other hand, the converse was the case for measurements of mandible sagittal position such as SNB, SNPg, FH^∧^NPg, and FH^∧^NB with *r* values ranging from 0.54 to 0.81. This result suggested that stronger sagittal relations between soft tissue and underlying hard tissue involved the lower third of the face compared with the middle third of the face.

Medium correlation coefficients were found for the vertical parameters (Table 4) in agreement with other studies [15]. Only SN^∧^FH exhibited low correlation with the respective soft tissue variables (*r* = 0.15 and *r* = 0.25). This may be because of the difficulty of locating corresponding soft tissue landmarks for the middle cranial base and the Go′ landmark that might have a small correspondence with the external soft tissues.

All the facial scans in the present study were performed with relaxed lips and this position was considered accurate in terms of diagnosis and treatment planning [4, 5] and allowed comparison with lateral cephalometric radiographs that are routinely performed with relaxed lips.

Unfortunately, the collected lateral cephalometric radiographs were not all performed with the same X-ray machine and this could be seen as a limitation of the present study. Moreover, only selected cephalometric landmarks were employed and only one operator analysed the data. Also, the inclusion of Caucasian patients only could be considered a limitation of the present investigation.

Even though encouraging results were obtained from the present study, they are still limited to our sample and methods. Also, the selected sample showed normal BMI, possibly the ideal condition for the present investigations since excessive BMI was reported to have significant effects on the ratio between skeleton and overlying soft tissue, so the results should not be extended to altered BMI conditions where the correspondences between hard and soft tissues may be less precise. Further studies are needed in order to clarify the complex relationships between soft and hard tissues and help clinicians and researchers with diagnosis and treatment planning with noninvasive tools.

## 5. Conclusions

From the results of the present study, the following facts can be stated:

- No statistically significant differences were found for sagittal and vertical soft tissue measurements between females and males in the tested sample.
- Sagittal measurements seemed to be more reliable in terms of providing a soft tissue diagnosis than lateral cephalometric radiography measurements (ANB and ANPg), especially for the lower third of the face (SNB, SNPg, FH^∧^NPg, and FH^∧^NB).
- Vertical soft tissue measurements showed weaker correlation with the corresponding lateral cephalometric radiography variables.

The present soft tissue analysis proposal based on 3D facial scans showed good reliability and reproducibility even though further studies are needed in order to confirm the findings of the research.

## Figures and Tables

**Table 1:** 2D and 3D cephalometric analyses. Cephalometric sagittal and vertical analyses and corresponding 3D soft tissue measurements.

Cephalometrics	3D soft tissue
Sagittal	
SNA	TrN′Ss
	ObsN′Ss
SNans	TrN′Sp
	ObsN′Sp
FH^∧^NA	TrOr^′∧^N′Ss
SNB	TrN′Sl
	ObsN′Sl
SNPg	TrN′Pg′
	ObsN′Pg′
FH^∧^NB	TrOr^′∧^N′Sl
FH^∧^NPg	TrOr^′∧^N′Pg′
ANB	SsN′Sl
ANPg	SsN′Pg′
FH^∧^AB	TrOr^′∧^SsSl

Vertical	
SN^∧^FH	TrN^′∧^TrOr′
	ObsN^′∧^TrOr′
FMA	TrOr^′∧^Go′Gn′
SN^∧^GoGn	TrN^′∧^Go′Gn′
	ObsN^′∧^Go′Gn′
Gonial s ArGoN	ObsGo′N′
Gonial i NGoMe	N′Go′Me′
Articulare SArGo	N′ObsGo′
NPg-GoMe	N′Pg^′∧^Go′Me′
SN^∧^ArGo	TrN^′∧^ObsGo′
	ObsN^′∧^ObsGo′
FH^∧^ArGo	TrOr^′∧^ObsGo′
FH^∧^SPg	TrOr^′∧^TrPg′
	TrOr^′∧^ObsPg′

**Table 2:** Sex differences. Data are shown as mean (in bold) and standard deviation (SD) (in italic) for the whole sample for females and for males. *P* values resulting from *t*-test to explore gender-specific differences are shown.

3D soft tissue	Mean female	SD female	Mean male	SD male	*P* value
Sagittal					
TrN′Ss	**79.75**	*2.53*	**81.46**	*3.41*	0.08
ObsN′Ss	**82.03**	*2.73*	**84.02**	*3.74*	0.06
TrN′Sp	**82.66**	*2.59*	**84.08**	*3.39*	0.12
ObsN′Sp	**84.94**	*2.43*	**86.63**	*3.83*	0.08
TrOr^′∧^N′Ss	**86.67**	*1.82*	**85.83**	*2.47*	0.17
TrN′Sl	**71.63**	*3.39*	**71.74**	*3.93*	0.47
ObsN′Sl	**73.91**	*3.48*	**74.30**	*4.29*	0.41
TrN′Pg′	**71.16**	*3.69*	**71.86**	*3.79*	0.33
ObsN′Pg′	**73.44**	*3.81*	**74.42**	*4.21*	0.28
TrOr^′∧^N′Sl	**84.24**	*3.63*	**83.92**	*3.53*	0.42
TrOr^′∧^N′Pg′	**83.71**	*3.97*	**84.02**	*3.38*	0.43
SsN′Sl	**8.11**	*1.99*	**9.72**	*2.43*	0.06
SsN′Pg′	**8.59**	*2.45*	**9.60**	*2.14*	0.17
TrOr^′∧^SsSl	**70.10**	*5.77*	**65.74**	*7.82*	0.06

Vertical					
TrN^′∧^TrOr′	**12.65**	*2.29*	**12.43**	*1.09*	0.41
ObsN^′∧^TrOr′	**10.41**	*3.06*	**9.68**	*1.47*	0.28
TrOr^′∧^Go′Gn′	**30.52**	*6.10*	**28.22**	*4.12*	0.18
TrN^′∧^Go′Gn′	**43.17**	*6.19*	**40.65**	*4.02*	0.16
ObsN^′∧^Go′Gn′	**40.92**	*6.41*	**37.91**	*3.72*	0.13
ObsGo′N′	**64.09**	*6.03*	**65.40**	*2.63*	0.29
N′Go′Me′	**70.19**	*5.84*	**70.36**	*5.51*	0.47
N′ObsGo′	**84.09**	*3.94*	**82.15**	*4.66*	0.14
N′Pg^′∧^Go′Me′	**65.72**	*4.63*	**67.76**	*6.46*	0.18
TrN^′∧^ObsGo′	**85.56**	*3.70*	**84.58**	*3.79*	0.27
ObsN^′∧^ObsGo′	**84.09**	*3.94*	**82.15**	*4.66*	0.14
TrOr^′∧^ObsGo′	**76.24**	*5.23*	**72.47**	*4.52*	0.05
TrOr^′∧^TrPg′	**38.31**	*2.85*	**36.62**	*1.86*	0.08
TrOr^′∧^ObsPg′	**44.47**	*3.01*	**43.99**	*1.80*	0.35

**Table 3:** Sagittal measurement correlations. Pearson correlation coefficients (*r*) are shown for sagittal measurements as low (in italic), medium (lightface), and high (in bold).  ^*∗*^ *P* < 0.05.

Cephalometrics			3D soft tissue			*r*
Variable	Mean	SD	Variable	Mean	SD	
SNA	81.43	3.35	TrN′Ss	80.08	2.81	0.36^**∗**^
			ObsN′Ss	82.41	3.05	0.34
SNans	85.55	3.84	TrN′Sp	82.94	2.82	0.31^**∗**^
			ObsN′Sp	85.27	2.84	*0.27*
FH^∧^NA	87.13	1.93	TrOr^′∧^N′Ss	86.51	1.99	*0.16* ^**∗**^
SNB	76.79	3.02	TrN′Sl	71.66	3.50	0.59^**∗**^
			ObsN′Sl	73.99	3.66	0.62^**∗**^
SNPg	77.47	3.02	TrN′Pg′	71.30	3.72	0.54^**∗**^
			ObsN′Pg′	73.63	3.91	0.56^**∗**^
FH^∧^NB	86.46	2.63	TrOr^′∧^N′Sl	84.17	3.61	0.67^**∗**^
FH^∧^NPg	86.70	2.20	TrOr^′∧^N′Pg′	83.77	3.87	0.66^**∗**^
ANB	4.65	2.36	SsN′Sl	8.42	2.18	0.74^**∗**^
ANPg	4.03	2.61	SsN′Pg′	8.79	2.43	0.74^**∗**^
FH^∧^AB	79.92	6.12	TrOr^′∧^SsSl	69.26	6.46	0.81^**∗**^

**Table 4:** Vertical measurement correlations. Pearson correlation coefficients (*r*) are shown for vertical measurements as low (in italic) and medium (lightface).  ^*∗*^ *P* < 0.05.

Cephalometrics			3D soft tissue			*r*
Variable	Mean	SD	Variable	Mean	SD	
SN^∧^FH	10.38	2.76	TrN^′∧^TrOr′	12.60	2.11	*0.25* ^*∗*^
			ObsN^′∧^TrOr′	10.26	2.84	*0.15* ^*∗*^
FMA	25.00	4.42	TrOr^′∧^Go′Gn′	30.07	5.84	0.59^*∗*^
SN^∧^GoGn	35.38	5.06	TrN^′∧^Go′Gn′	42.68	5.92	0.54^*∗*^
			ObsN^′∧^Go′Gn′	40.34	6.10	0.53^*∗*^
Gonial s ArGoN	53.46	4.06	ObsGo′N′	64.35	5.56	0.42^*∗*^
Gonial i NGoMe	75.66	4.56	N′Go′Me′	70.22	5.78	0.45^*∗*^
Articular SArGo	142.06	6.62	N′ObsGo′	83.72	4.16	0.45^*∗*^
NPg-GoMe	67.15	4.28	N′Pg^′∧^Go′Me′	66.12	5.10	0.61^*∗*^
SN^∧^ArGo	84.69	3.97	TrN^′∧^ObsGo′	85.37	3.74	0.33
			ObsN^′∧^ObsGo′	83.72	4.16	0.43
FH^∧^ArGo	75.87	5.38	TrOr^′∧^ObsGo′	75.51	5.32	0.45
FH^∧^SPg	57.16	3.13	TrOr^′∧^TrPg′	37.98	2.77	0.57
			TrOr^′∧^ObsPg′	44.38	2.82	0.59
